# Localization of lung abnormalities on chest X-rays using self-supervised equivariant attention

**DOI:** 10.1007/s13534-022-00249-5

**Published:** 2022-11-03

**Authors:** Gavin D’Souza, N. V. Subba Reddy, K. N. Manjunath

**Affiliations:** 1 Department of Instrumentation and Control Engineering, Manipal Institute of Technology, Manipal Academy of Higher Education, Manipal, Karnataka 576104 India; 2 Department of Information Technology, Manipal Institute of Technology Bengaluru, Manipal Academy of Higher Education, Manipal, Karnataka 560064 India; 3 Department of Computer Science and Engineering, Manipal Institute of Technology, Manipal Academy of Higher Education, Manipal, Karnataka 576104 India

**Keywords:** Self-supervised equivariant attention, ResNet50, Siamese network, Weak supervision, Pixel correlation module, Self-attention, CAM

## Abstract

Chest X-Ray (CXR) images provide most anatomical details and the abnormalities on a 2D plane. Therefore, a 2D view of the 3D anatomy is sometimes sufficient for the initial diagnosis. However, close to fourteen commonly occurring diseases are sometimes difficult to identify by visually inspecting the images. Therefore, there is a drift toward developing computer-aided assistive systems to help radiologists. This paper proposes a deep learning model for the classification and localization of chest diseases by using image-level annotations. The model consists of a modified Resnet50 backbone for extracting feature corpus from the images, a classifier, and a pixel correlation module (PCM). During PCM training, the network is a weight-shared siamese architecture where the first branch applies the affine transform to the image before feeding to the network, while the second applies the same transform to the network output. The method was evaluated on CXR from the clinical center in the ratio of 70:20 for training and testing. The model was developed and tested using the cloud computing platform Google Colaboratory (NVidia Tesla P100 GPU, 16 GB of RAM). A radiologist subjectively validated the results. Our model trained with the configurations mentioned in this paper outperformed benchmark results.

**Supplementary Information:**

The online version contains supplementary material available at 10.1007/s13534-022-00249-5.

## Introduction

**Fig. 1:**
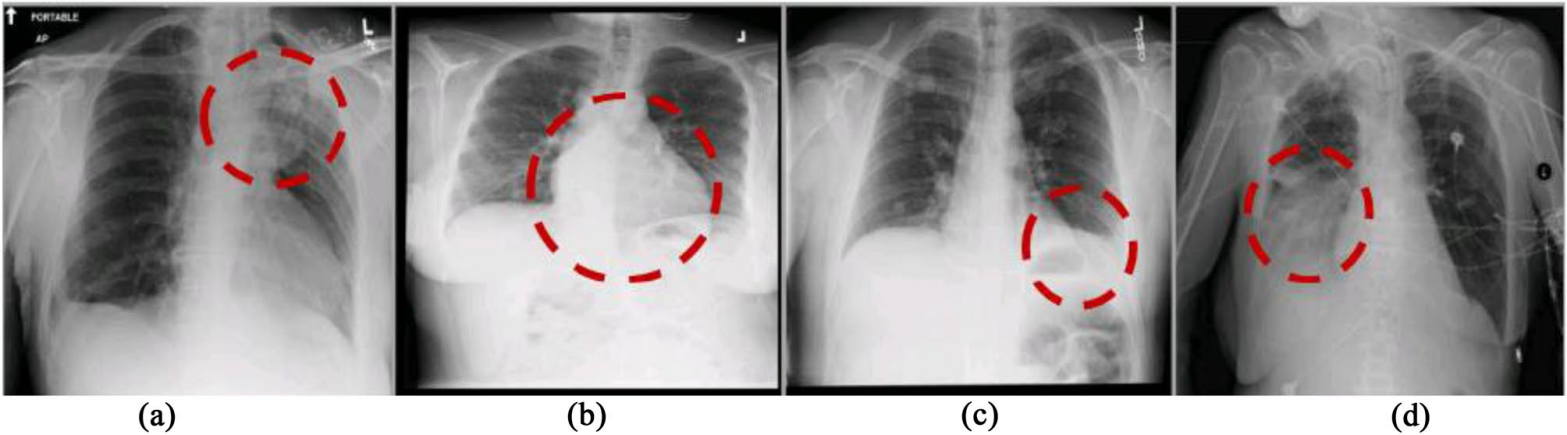
Lung diseases and their appearance on anterior to posterior CXR pose a great challenge to validate in a CAD system (images from [1]). Few examples, **a** Atelatasis, **b** cardiomegaly, **c** effusion, **d** Infiltration

Chest X-Ray (CXR) radiographs show the anatomical structure and the pathology in the R^2^ dimension. Clinically the diagnosis is more challenging than even the CT modality. Reading and analyzing an X-Ray often requires experience and knowledge of anatomical principles, physiology, and pathology. This modality is widely used for the lungs as the first screening level, and further CT/MRI is done to know the volumetric details of anatomies. The commonly occurring diseases (14 types) in the chest are, *Pleural_Thickening*, *Fibrosis, Atelectasis, Pneumothorax, Mass, Pneumonia, Nodule, Cardiomegaly, Edema, Consolidation, Effusion, Emphysema, Infiltration,* and *Hernia* [1] (Fig. 1)*.* With many patients visiting the radiology centers, it is time-consuming to go through every image for an accurate diagnosis in any of these cases.

Recently Deep Learning (DL) algorithms have led to rapid advances in computer vision and medical imaging. It is finding its place in radiology centers as a supporting and assisting tool for radiologists. DL has dominated medical imaging for many tasks such as classification, detection, segmentation, etc. When trained on large datasets, these algorithms give accurate results and have shown scalable performance with increased training data. A drawback of these algorithms is that they are highly data-hungry and require large-scale labeled datasets. Large-scale labeling by an expert radiologist involves lots of effort and time. Localization tasks like object detection and semantic segmentation require strong supervision of bounding boxes pixel-wise labels. Researchers have developed a DL model to classify Tuberculosis (TB) [2]. However, their computer-assisted diagnosis (CAD) was not clinically validated as only a few thousand images were employed in the study. This is evident from [3], where the performance of deep neural networks for thorax disease recognition was limited by the availability of only 4143 AP images [4] (as of now, OpenI was the largest publicly available database for CXR).

Weakly supervised techniques such as CAM [1, 3, 4] have demonstrated the localization ability of CNN classifiers trained with purely image-level labels. However, these CAMs only provide a rough estimate of the most discriminate part of the object (under-activation) and incorrectly activate in the background region of the image (over-activation), when they are rescaled back to the original image size, due to the loss of spatial information at each stage of the CNN model. Therefore, equivariant regularization [5] is employed to introduce an additional source of supervision. All results are evaluated on the fourteen datasets.

## Literature review

In this section, various DL models for CXR analysis are discussed. Fourteen CXR datasets were first introduced by [1], with 1,12,120 anterior to posterior X-rays from 30,805 patients. This dataset covers 14 chest diseases (mentioned in the introduction), of which 60,412 cases are without *pathology* and 51,708 have one or more pathologies. It was demonstrated that thoracic diseases could be accurately localized using a weakly supervised multi-label learning framework. A variety of CNN architectures were tested, including Alexnet [6], GoogLeNet [7], VGG16 [8], and Resnet50 [9], among which the Resnet50 showed superior performance in both classification and localization tasks. A CNN model (121-layer) named CheXNet with DenseNet architecture was discussed in [3, 4]. The model takes CXR as inputs and produces the class probabilities and a heatmap [10] as output, indicating the most activated region in the image for each class of diseases. It used pre-trained weights on the ImageNet dataset and outperformed the previous methods [1, 5]. In [11], the author trained a multi-class classifier to classify 14 diseases using ResNet50 as base architecture. They studied the effects of transfer learning on the model performance with and without fine-tuning and training the model from scratch. Fine-tuning and training from scratch showed similar overall performance, whereas an off-the-shelf model underperformed significantly. They also demonstrated an increase in performance in incorporating the clinical features. GradCAM was used to generate heat maps.

In [1, 3, 11], the authors have used *unweighted global pooling* operations to generate image-level feature vectors accepted by the classification layer. However, this results in the model learning highly over-activated or under-activated feature maps since pooling operations make the model focus more on local features [12]. On the other hand, Self-attention [12, 13] is proven to be capable of focusing on long-range information. Therefore, it is preferable to use a combination of global pooling and self-attention to learn CAM generation. A modified DenseNet121 network was discussed in [14]. It was equipped with SE [15] blocks between consecutive dense blocks followed by a classwise multi-map transfer layer and max–min pooling for classifying thoracic diseases and detecting lesions. The SE blocks serve as an attention mechanism [12] as it recalibrates the output feature maps according to their global distribution on the feature channel. The multi-map layer encodes the activation outputs of the backbone network into M individual feature maps for each disease class through a 1 × 1 convolution operation. As a result, the model outperformed previous methods [1, 16]. [16–18] proposed multiple instance learning techniques for CXR classification and localization. Multi-instance learning (MIL) is a supervised method with a single class label is assigned to a bag of instances. [17] proposed an attention-based MIL that uses a weighted average of instances to compute a bag-level representation. A neural network computes the weights. A classifier is trained to classify the bag-level representation. The weights corresponding to each instance are used to generate a heatmap that detects key instances. [18] proposed a new probabilistic global pooling operation that explicitly leveraged CAM for localization during training. The model outperformed the LSE (Log Sum Exponential) pooling baseline in [1].

Localizing diseases on CXR is challenging as it often requires identifying anomalies of varying sizes. Although the pooling operations within the backbone network improve computational efficiency, they lead to a loss in spatial information. This decreases the accuracy of the generated heatmaps. [19–21] propose methods to include intermediate feature maps at various stages in the backbone network when computing the output feature maps. These intermediate characteristic maps enable the model to integrate information from multiple scales and abstraction levels. [19] uses a ResNet backbone to process images and generate multi-scale feature maps combined sequentially in a coarse (low resolution) to acceptable (high resolution) manner using upsampling and DenseNet blocks. A dense connection is applied per resolution scale, followed by upsampling and channel-wise concatenation fuse information from multiple resolutions. Finally, LSE-LBA pooling aggregates instance scores and outputs the global probability. The dataset was split into 70% training set, 10% validation set, and 20% for testing. The model achieved an AUC = 87% and IoU = 63% for pneumothorax detection and localization.

The CAM [10] is an effective way to localize objects using image-level classification labels. However, these CAMs are prone to over-activating in the background regions or under-activating in object regions. This is due to the supervision gap between fully and weakly supervised learning. [22] proposed a method to reduce this gap in supervision by leveraging the inconsistencies in CAMs when images are subjected to affine transformations. A self-supervised equivariant attention mechanism (SEAM) was discussed, incorporating equivariant regularization with pixel correlation module (PCM), which further refined generated CAMs using an attention mechanism. The model was trained on the PASCAL VOC 2012 dataset and achieved a mIoU of 55.41%, surpassing the performance of GradCAM and GradCAM +  + .

## Materials and methods

The required CXR were downloaded from the Clinical Center of the National Institute of Health (NIH) [1, 23, 24]. The server has nearly 60% of all hospitals' anterior to posterior (AP) CXR. Dataset poses realistic clinical diagnosis challenges. The dataset for training the model consists of 112,120 AP images (in PNG format) from 30,805 subjects. Each image has multi-labels (14 different disease labels as listed in the introduction). The database size and the thorax disease frequencies facilitate good DL model training. The radiology reports were unavailable due to the data protection policy. Each image has metadata, but it was not considered for training. About 1000 images have been annotated with bounding boxes. Images were resized to a resolution of $1024 \times 1024$. The annotations contain the image index, disease finding a label, and the bounding box Bbox [$x, y, h, w$], where [$x, y$] are coordinates of the top left corner and [$h, w$] correspond to the height and width of each box. Further, two data split files containing the names of all images meant for training and testing, respectively. Images in the CXR dataset are divided into these two sets at the patient level. All studies from the same patient will only appear in either training, validation, or testing set. Some cases are there with follow-up studies also. The dataset was first released in 2017 as annotated images and the bounding box details in the training set (train_val_list.txt) and the test set (test_list.txt). PyTorch libraries and associated python packages were used in the google colab cloud environment in this work for programming.

Upon publication, the DL model code and the necessary steps to reproduce the output will be published to the scientific community through the GitHub repository to reach a wider audience. Other researchers can use this model to assess model output qualitatively. This helps to reuse the existing code, reuse data, validate the accuracy of our results, develop new solutions, and increase the performance of R&I (the instructions to reproduce the work are available in the supplementary material).

## Methodology

### ResNet backbone

**Fig. 2:**
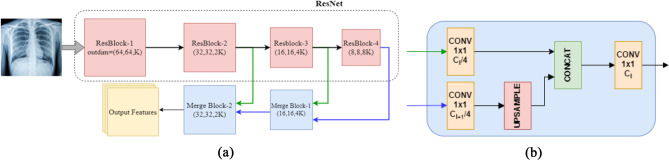
**a** Multi-resolution ResNet backbone architecture that sequentially reintegrates spatial information from previous layer outputs using the Merge Block. **b** shows the internal structure of the Merge Block which uses upsampling and concatenation to merge feature maps.

Here a modified version of the Multi-Resolution (MR) ResNet model [19] is proposed and adopted as a backbone feature extractor to extract the feature corpus from the CXR. The feature extractor uses a pre-trained (on ImageNet dataset) ResNet50 as its base. While coarse-scale features extracted by the traditional ResNet model are ideal for the classification task, they lack the spatial information needed to compute high-resolution CAMs. For example, in Resnet50, for an image size of $256 \times 256$, the dimensions of the resulting CAM is $8 \times 8$*,* which lacks the spatial information needed to upsample to $256 \times 256$ accurately. Therefore, to have accurate CAMs it is necessary to maintain the spatial information of the feature maps. Fine-scaled, higher resolution features extracted by initial layers capture detailed spatial information. The proposed network shown in Fig. 2a reintegrates these higher-resolution features from earlier layers in the network back into the coarse output feature representations to produce resolution-preserving feature maps. This is done in a coarse to the fine manner by repeating Eq. 1.

1 $$F_{o}^{l} = g\left( {F_{o}^{l + 1} ,F^{l} } \right)$$

where $F_{o}^{l}$ denotes the resolution-preserving feature map at resolution level $l$, $F_{o}^{l + 1}$, the previously computed resolution-preserving feature map at lower resolution level $l$ + 1, $F^{l}$ the feature map at resolution level $l$ obtained directly from the network. $g$ denotes the Merge Block. Figure 2b shows the structure of the Merge Block, wherein the channel lengths of $F_{o}^{l + 1}$ and $F^{l}$ are each reduced by a factor of 4 by 1 × 1 Conv layers followed by ReLU activation. Next, bilinear interpolation is used for upsampling the spatial dimensions of $F_{o}^{l + 1}$ to match that of $F^{l}$. Finally, the feature maps are concatenated, and a 1 × 1 Conv layer (with ReLU activation) resizes the channel length of the concatenated feature maps to match that of the original $F^{l}$.

The feature extractor network extracts output feature maps that contain both the semantic and spatial information required for the classification and CAM generation tasks. Log-Sum-Exponential pooling (LSE) [1] is employed to compute the final feature vector, fed to the classification layer to compute the prediction vector $z$. The classification layer also computes CAMs by feed-forwarding the output feature maps without global pooling. Generated CAMs are further refined using the Pixel Correlation Module (PCM) [22]. Before PCM refinement, non-maximum activations in the CAMs are suppressed to zero. Note that PCM refinement does not affect the model’s classification task. The backbone feature extractor and classification layer are fine-tuned on the Chest-Xray14 dataset using a dynamically weighted multi-label BCE loss (Sect. 4.3).

### Pixel-correlation module (PCM)

**Fig. 3:**
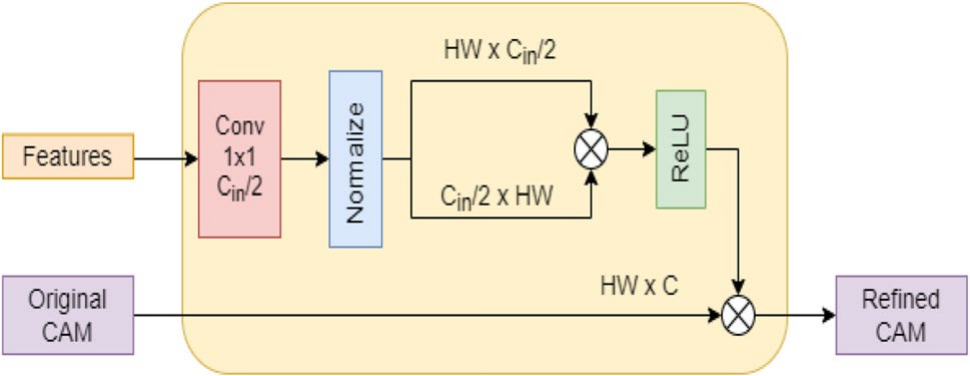
Structure of PCM. H, W, $C_{in}$ /$C$ denote the height, width and channel length of feature maps and original cams

As shown in Fig. 3, the PCM employs modified self-attention to refine the pixel-wise CAM predictions by using context information from feature maps produced by the backbone feature extractor [22]. For a single pixel, PCM refinement is applied as follows,

2 $$y_{i} = \frac{1}{{C\left( {x_{i} } \right)}}\sum\limits_{{\forall j}} {} \quad ReLU\left( {\frac{{\theta \left( {x_{i} } \right)^{T} \theta \left( {x_{j} } \right)}}{{||\theta \left( {x_{i} } \right)|| \cdot {\text{ ||}}\theta \left( {x_{j} } \right)||}}} \right)\hat{y}_{j}$$

Here, $x$ denotes the features extracted by the backbone feature extractor, $\hat{y}$ denotes the original CAM, $y$ denotes the revised CAM and $\theta$ is an embedding function implemented by a 1 × 1 Conv layer without non-linear activation. Inner product in normalized feature space of the embeddings is used to calculate the affinity between current pixel $i$ and every other pixel $j$. The similarities are then activated using ReLU activation to suppress negative values. Each pixel in the final CAM is the weighted average of all pixels in the original CAM; wherein the normalized similarities serve as the weights.

### Multi-label BCE loss

Due to a large imbalance in the number of positive and negative labels, models trained with standard BCE Loss on the Chest-Xray 14 dataset tend to largely overfit and produce a constant negative (“0”) output. To reduce the effects of the class imbalance, the standard BCE loss is weighted dynamically. As such, the modified BCE loss function is as follows,

3 $$l\left( {X,Y} \right) = \mathop \sum \limits_{c = 0}^{n} \left( { - \beta_{P}^{c} \cdot y_{c} \cdot log\left( {f\left( {x_{c} } \right)} \right) - \beta_{N}^{c} \cdot \left( {1 - y_{c} } \right) \cdot log\left( {1 - f\left( {x_{c} } \right)} \right)} \right)$$

Here, $c$ refers to a specific class, $n$ is the total number of unique class labels, $\beta_{P}^{c}$ and $\beta_{N}^{c}$ are class-specific weights that are dynamically computed for every batch as

4 $$\beta _{P}^{c} = \frac{{\left| {P_{c} } \right| + \left| {N_{c} } \right|}}{{\left| {P_{c} } \right|}}$$

5 $$\beta _{N}^{c} = \frac{{\left| {P_{c} } \right| + \left| {N_{c} } \right|}}{{\left| {N_{c} } \right|}}$$

where $P_{c}$ and $N_{c}$ are the total number of positive and negative samples in the batch belonging to class $c$.

### PCM training using self-supervised equivariant attention mechanism (SEAM)

**Fig. 4:**
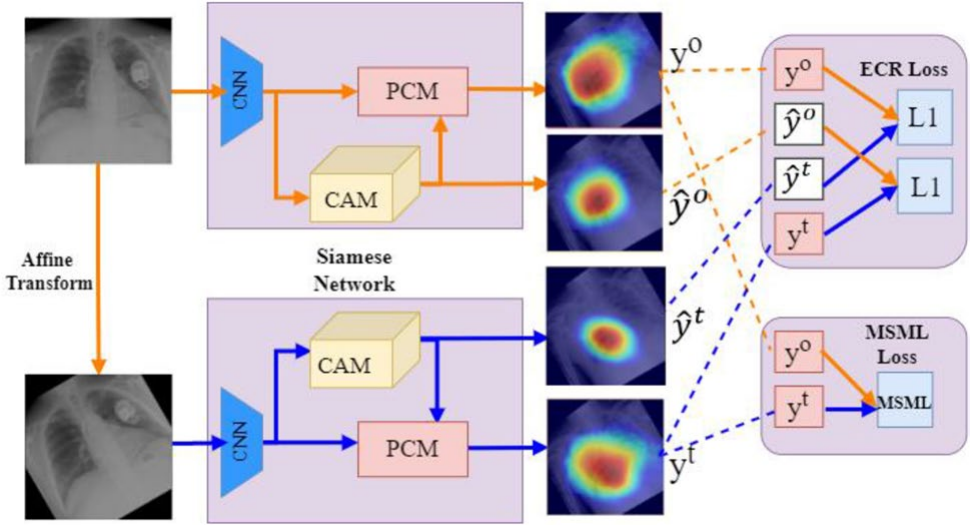
Siamese Network for SEAM during PCM training. PCM refines the pixel-wise CAM predictions by using context information from feature maps produced by the backbone feature extractor. Image augmentations by affine transformations create inconsistencies in the generated CAMs compared to the original CAMs. Equivariant regularization enables the use of these inconsistencies for PCM training. MSML loss provides an additional source of supervision

The PCM model is trained separately from the feature extractor and classifier. In this section, the training process for PCM using SEAM is described. During PCM training, the weights of the feature extractor and classifier are frozen. It is trained using image-level labels as the only source of human-annotated supervision. Image augmentation by affine transformations creates inconsistencies in the generated CAMs compared to the original CAMs. Applying consistency regularization to these augmented images provides a source of self-supervision for training. SEAM is the integration of PCM and equivariant regularization [22]. As shown in Fig. 4, During PCM training, the network is a weight-shared Siamese architecture where one branch applies the affine transform to the image before feeding it to the network, while the other applies the same transform to the network output. The PCM is trained using Equivariant Cross Regularization (ECR) proposed by [22] as a source of supervision. ECR loss is given by,

6 $$loss_{{ECR}} = {\text{ ||}}A\left( {y^{o} } \right) - \hat{y}^{t} ||_{1} - {\text{ ||}}A\left( {\hat{y}^{o} } \right) - y^{t} {\text{ ||}}_{1}$$

Here *A* is the affine transform. $\hat{y}^{o}$ and $\hat{y}^{t}$ refer to the original CAMs of the siamese network from the branch with an original and transformed image input, respectively. Similarly, $y^{o}$ and $y^{t}$ refer to the CAMs refined by PCM. Online hard example mining (OHEM) is applied to $loss_{ECR}$ to keep only the top 20% largest pixel losses. The affine transformations used to compute the pseudo labels in SEAM Rescale include only random rotation between (-30° to 30°). We do not consider flip transforms as there is no significant difference in performance. Further, as an added source of supervision for PCM training, we apply global average pooling to the refined feature maps in both branches of the Siamese network to produce prediction vectors and compute the multi-label soft-margin loss ($Loss_{MSML}$) using the ground truth class labels.

The net loss value is computed as,

7 $$loss_{PCM} = \lambda_{MSML} *Loss_{MSML} + \lambda_{ECR} *Loss_{ECR}$$

where, $$\lambda_{MSML} =$$ 1.5 and $$\lambda_{ECR} = 1$$.

## Results and discussion

### Implementation

**Fig. 5:**
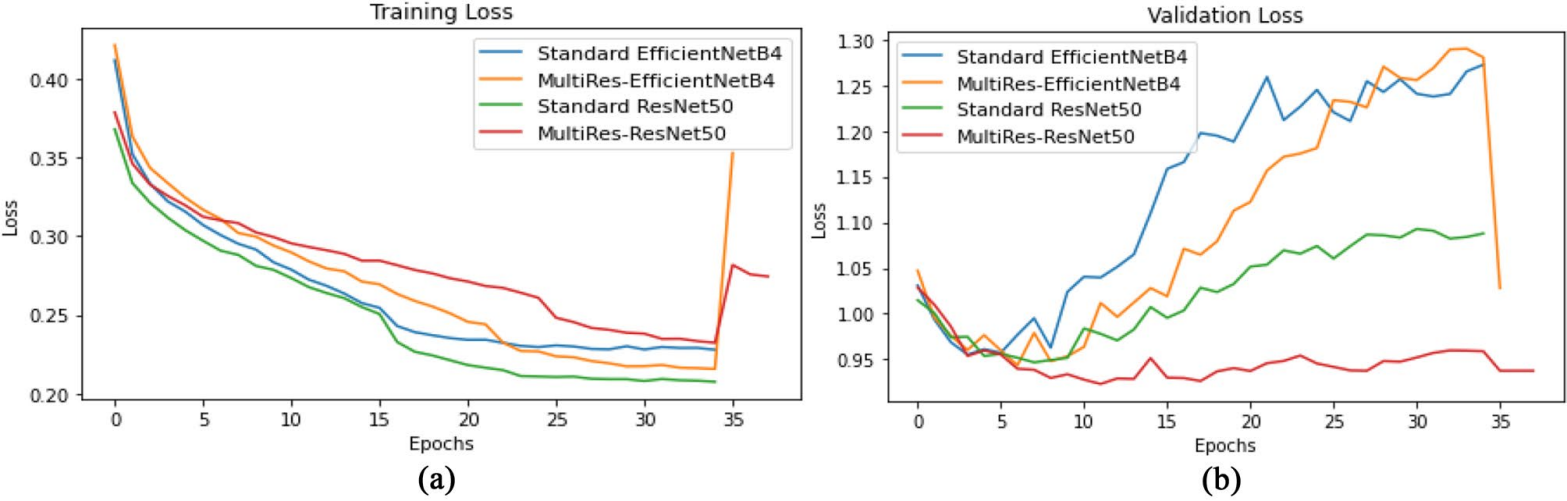
**a** Shows the training loss curves during training for all models. **b** Shows the validation loss curves during training for all models.

ResNet50 pre-trained on ImageNet is employed as the backbone network, with only the third and fourth ResNet blocks made trainable. Fine-tuning earlier layers of the pre-trained backbone network quickly led to overfitting. A standard ResNet50 is trained and evaluated. Further, EfficientNetB4 is employed as a backbone to test the performance of the methods. All models use LSE pooling with γ = 10 to compute class prediction vectors for the classification task. For feature extractor and classifier training, Adam optimizer with parameters with beta values as (0.9,0.9) is employed with the learning rate set to 3E-04. The feature extractor is trained for 35 epochs. Similarly, for PCM training using SEAM losses, Adam optimizer was used with a learning rate set to 1E-06 for 3 epochs. For PCM training with MultiRes-EfficientNetB4 backbone, learning rate of 1E-05 and 1 epoch of training gave best performance. In all cases, Gradient accumulation enables training with larger batch sizes. A batch size of 32 is used with 3 gradient accumulation steps, resulting in a batch size of 96 during weight updates. All models were trained and tested using Nvidia Tesla P100 GPU with 16 GB memory using the cloud computing platform Google Colaboratory. To cross-check our results with the clinical notes, we could not do as the radiological reports were not available along with the image from NCI. Also, to cross-check the training and testing on the network discussed in [1], the code for the pre-trained model was not released. In the results, if there are no findings, the listed 14 disease patterns are not found in the image. A radiologist compared the heatmap results with results published in [1]. PyTorch libraries and associated python packages were used in the google colab cloud environment in this work. Figure 5 shows the training and validation loss curves for all models. During epochs 0–34 the backbone feature extractor and classifier are trained using BCE loss. The sudden increase or decrease in loss value at epoch 35 is due to PCM training using SEAM.

### Dataset classification

The method was evaluated on CXR from the clinical center [1]. The data set contains 112,120 images. The official test set consists of 25,596 (~ 22%) images. The remaining 86,524 comprise 50,500 cases that do not contain any disease. We use all images associated with any disease and randomly select approx. 5000 disease-free images. To reduce the class imbalance of disease-free images. The dataset for training and validation was split in to 80:20, respectively. During training, we resize all images to 256 $\times$ 256 and randomly sample various sized patches with sizes between 80 and 100% within the image area. Before resizing, the random aspect ratio of the crop varies between 0.75 and 1.733. Images were also augmented using the random horizontal flip and random rotation of the image (-15° to 15°). The images are normalized via the mean and standard deviation of the ImageNet dataset.

**Fig. 6:**
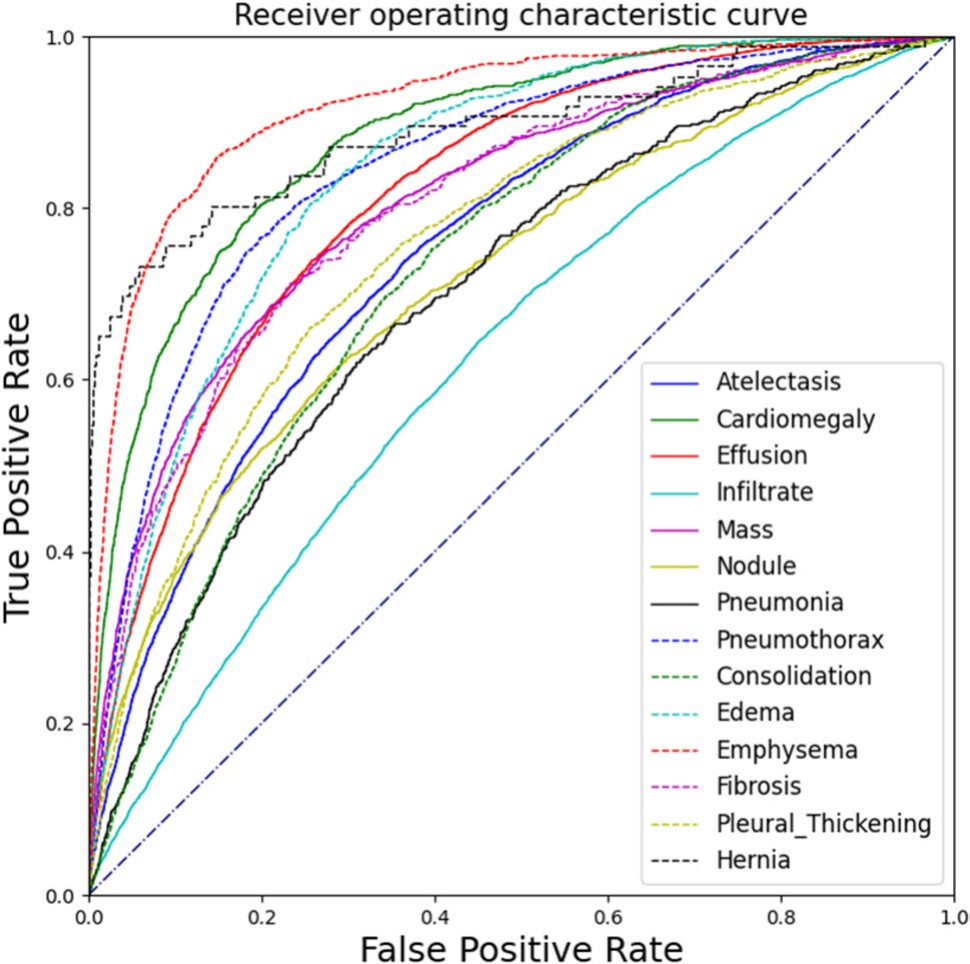
ROC Curve for the performance of MultiResolution-ResNet50

As shown in Table 1, the standard ResNet50 baseline model trained with the configurations mentioned in this paper outperformed benchmarks set in [1]. Similarly, our MultiRes-ResNet50 and MultiRes-EfficientNetB4 models outperform previous works [1, 19]. The Multi-Resolution ResNet50 and EfficientNetB4 models have classification performance similar to their standard counterparts. For the classification task, ResNet50 models are superior to EfficientB4 models. ROC curve is plotted for the MultiRes-ResNet50 model (Fig. 6). Our model gives competitive classification performance with newer state-of-the-art methods such as [25]. However, on average, it does not outperform [25] and in the case of resnet50 the difference in performance was less than 2%. However, we could not make an accurate comparison to [25] model as they used a unique image resampling scheme involving patient metadata to construct a balanced dataset to mitigate class imbalance. This work uses the official test train split, wherein a portion of the train set is used for validation. Class imbalance is handled entirely by the loss function during training. The models and training methods used in this study show improvements in performance compared to previous studies [1, 19] that are based on the same standard resnet50 model. Similarly, the modified multi-resolution model outperforms in [19] who used a model of a similar scale with high-resolution input images (512 $\times$ 512) and more Conv and upsampling layers. Also, [19] uses a unique pooling operation called Log-Sum-Exp Pooling with Lower-bounded Adaptation (LSE-LBA) as the global pooling operation to improve performance. Our model uses the regular LSE pooing operation (also used in [1]).

**Table 1:** Classification AUCs of all 14 diseases in the Chest x-ray 14 test set

Pathology	Standard ResNet50 [1]	Multi-resolution ResNet50 [19]	MobileNetV2 [25]	Standard Resnet50 (our version)	MultiRes-ResNet50 (ours)	Standard Efficientnetb4 (our version)	MultiRes-EfficientNetB4 (ours)
Atelectasis	0.700	0.733	0.794	0.751	0.752	0.738	0.743
Cardiomegaly	0.810	0.856	0.885	0.868	0.883	0.863	0.845
Effusion	0.759	0.806	0.876	0.817	0.817	0.809	0.811
Infiltration	0.661	0.673	0.711	0.633	0.627	0.643	0.617
Mass	0.693	0.777	0.826	0.796	0.809	0.762	0.763
Nodule	0.669	0.718	0.743	0.732	0.718	0.711	0.704
Pneumonia	0.658	0.684	0.733	0.705	0.701	0.659	0.673
Pneumothorax	0.799	0.805	0.880	0.844	0.848	0.834	0.839
Consolidation	0.703	0.711	0.790	0.734	0.730	0.725	0.729
Edema	0.805	0.806	0.884	0.845	0.844	0.835	0.828
Emphysema	0.833	0.842	0.891	0.925	0.920	0.862	0.862
Fibrosis	0.786	0.743	0.762	0.822	0.806	0.816	0.793
Pleural thickening	0.684	0.724	0.763	0.739	0.761	0.741	0.726
Hernia	0.872	0.775	0.811	0.935	0.891	0.931	0.910
Mean	0.738	0.761	0.810	0.796	0.793	0.781	0.774

### Localization task

**Fig. 7:**
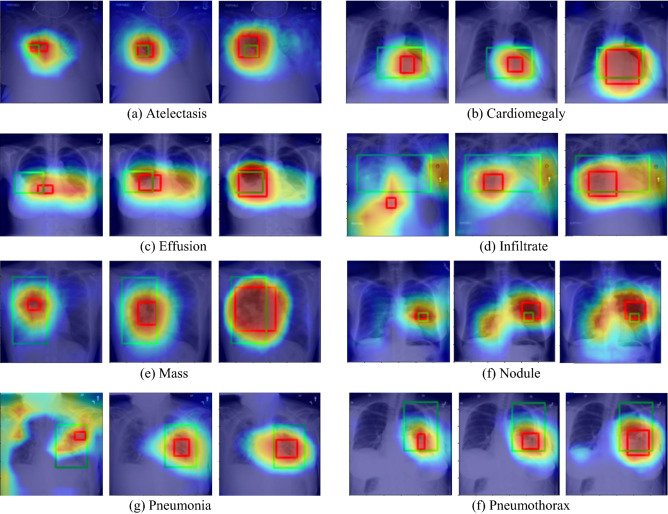
Sample localization heatmaps and bounding boxes generated by standard ResNet50 (left), MultiRes-ResNet50 (Middle), and MultiRes-ResNet50 CAMs refined using PCM (right). In each figure, green bounding boxes indicate ground truths, whereas red bounding boxes indicate predicted predictions by the corresponding model

CAMs are used to generate heatmaps for the localization task. Classwise bounding boxes are computed by applying binary thresholding to each heatmap with a constant threshold Q = 0.7, followed by a contour detection algorithm in OpenCV. The CAMs are then scaled to be in the range [0,255] and color-mapped. The model's localization performance is evaluated on eight classes for which human-annotated ground truth bounding boxes are available. Intersection over the predicted B-Box area ratio (IoBB) measures the overlap between predicted and ground truth bounding boxes [1]. When at least one predicted bounding box is overlapped with the ground truth (IoBB > T(IoBB)), then we define it as correct localization. Finally, we compare the localization performance of Multi-resolution models to the standard baselines. As such, we compute the classwise localization accuracy (Lacc) and Average False Positive Number (FPN) similar to [1] (Table 2 and Table 3). Heatmaps are shown in Fig. 7.

**Table 2:** Localization accuracies of the eight diseases which have bounding box annotations in the ChestX-ray14 test set

Method	Atelectasis	Cardiomegaly	Effusion	Infiltration	Mass	Nodule	Pneumonia	Pneumothorax
Localization accuracy, IoBB = 0.1								
Standard ResNet50	0.428	0.788	0.510	0.220	0.365	0.114	0.242	0.143
MultiRes-ResNet50	0.378	**0.829**	0.588	0.122	0.518	**0.241**	0.208	**0.173**
MultiRes-ResNet50 (with PCM)	0.450	**0.829**	**0.686**	0.195	**0.553**	0.177	**0.275**	**0.173**
Standard EfficientNetB4	0.456	0.795	0.680	0.374	0.271	0.000	0.108	0.204
MultiRes-EfficientNetB4	**0.506**	0.699	0.660	**0.496**	0.435	0.203	0.267	**0.214**
MultiRes-EfficientNetB4 (with PCM)	0.278	0.685	0.601	0.390	0.365	0.038	0.242	0.204

The values in bold signify the least false positive numbers

**Table 3:** Average false positive numbers of the eight diseases which have bounding box annotations in the ChestX-ray14 test set

Method	Atelectasis	Cardiomegaly	Effusion	Infiltration	Mass	Nodule	Pneumonia	Pneumothorax
Standard ResNet50	0.580	0.115	**0.591**	**0.615**	**0.276**	0.550	0.361	0.355
MultiRes-ResNet50	0.753	0.130	0.817	0.969	0.360	0.430	0.311	0.384
MultiRes-ResNet50 (with PCM)	0.467	0.106	0.686	1.103	0.292	0.278	0.228	0.302
Standard EfficientNetB4	0.720	1.180	1.018	0.903	0.848	0.773	0.332	1.434
MultiRes-EfficientNetB4	**0.443**	**0.091**	0.600	0.755	0.300	**0.251**	**0.199**	**0.281**
MultiRes-EfficientNetB4 (with PCM)	0.762	0.394	0.984	0.767	0.403	0.384	0.241	0.331

The values in bold signify the least false positive numbers

The MultiRes-ResNet50 (Without PCM) and MultiRes-EfficientNetB4 (Without PCM) give a higher Lacc than their respective standard counterparts (see Table 2). The multi-resolution backbone produces a higher resolution CAM that preserves spatial information. This CAM can then be accurately rescaled to the original image dimensions using bilinear interpolation, compared to CAMs produced by standard models, wherein, when rescaled, the activation sometimes does not overlap with the actual abnormality region as seen in Fig. 7. This indicates the lower Lacc of the standard models is due to the lack of spatial information in low-resolution CAMs that are directly rescaled to the resolution of the input image and that reintegrating higher resolution features from the initial layers of the model improves the localization performance. Further, the PCM module refines the CAMs using global attention. It acts as a trainable filtering operation that expands and sharpens the boundaries of activated regions and reduces the number of false positive bounding boxes created by the thresholding and contour detection algorithms in OpenCV. MultiRes-EfficientNetB4 has a lower FPN than its standard counterpart while FPN in MultiRes-ResNet50 is more significant than in its standard counterpart. In MultiRes-ResNet50, PCM refinement increases the Lacc in 5 classes and decreases the FPN in 7 classes. In particular, we see large improvements in the localization of Atelectasis, Effusion, Infiltration, and Pneumonia. Overall, the combined Multires-ResNet50 (with PCM) gives better localization performance than the Multires-ResNet50 (without PCM) and standard ResNet50. PCM refinement in MultiRes-EfficientNetB4, however, tends to degrade localization performance. For EfficientNetB4, the Multires-EfficientNetB4 (without PCM) gave better localization performance compared to standard efficientNetB4 in most classes (see Table 2).

### Inference speed

Table 4 shows the inference times and number of parameters of each model. The Inference times were measured for images having dimensions $256 \times 256 \times 3$ on a single NVidia P100 GPU. Note that ResNet50 is computationally more efficient than EfficientNetB4.

**Table 4:** Inference times for various models

Model	Inference time (ms)	Number of parameters
Standard ResNet50	15.51	23,536,718
MultiRes-ResNet50	16.93	26,273,615
Standard EfficientNetB4	20.29	17,573,718
MultiRes-EfficientNetB4	23.46	18,913,543

## Conclusion

We have discussed a weakly supervised deep learning technique for the simultaneous classification and localization of lung abnormalities using CXR. A modified Resnet50 for extracting the feature corpus from the images, a classifier, and a pixel correlation module for refining CAMs were included. The method was applied to the largest clinical data published by Ronald Summers (NIH, USA). The model performance was compared with the published results on this dataset. Our classification accuracy is slightly improved and better than the existing publication for the same base model architecture (ResNet50). We show PCM's effectiveness in improving the model's disease localization ability. The scope of future work is to exploit more radiomics features from the images, which helps for better classification and predictions. Weakly supervised multiple instance learning techniques such as probabilistic CAM pooling (PCAM) can be explored to improve CAM accuracy. Patch-wise, Self-attention mechanisms such as multi-head self-attention (MSA) that have shown impressive results in computer-vision applications can be leveraged for CAM refinement instead of PCM.

## Supplementary Information

Below is the link to the electronic supplementary material.

Supplementary file1 (DOCX 19 kb)
