# Supplementary material for: Localization of lung abnormalities on chest X-rays using self-supervised equivariant attention
Source: Biomed Eng Lett. 2022 Nov 3;13(1):21–30. doi: 10.1007/s13534-022-00249-5 (PMC9873849; doi:10.1007/s13534-022-00249-5)
Supplement: Supplementary file 1 — Supplementary file1 (DOCX 19 kb) [file 13534_2022_249_MOESM1_ESM.docx]

**Localization of Lung abnormalities on Chest X-Rays using self-supervised equivariant attention**

**Steps to reproduce the DL model output**

| **01.** | **Check GPU configuration** |
| --- | --- |
|  | !nvidia**-**smi |
|  |  |
| **02.** | **Clone the Repository** |
|  | !git clone https:**//**github**.**com**/**gavin**-**d26**/**Localization**-**of**-**Lung**-**abnormalities**-**on**-**Chest**-**X**-**Rays**-**using**-**self**-**supervised**-**equivariant**-**attention**.**git |
|  |  |
| **03.** | **Create a kaggle API token and upload the downloaded kaggle.json to the content directory** |
|  | from google.colab import files  files.upload() |
|  |  |
| **04.** | **Create a directory named kaggle that will store the kaggle.json file, and display list of kaggle datasets.** |
|  | !mkdir **~/.**kaggle  !mv **./**kaggle**.**json **~/.**kaggle**/**  !chmod 600 **~/.**kaggle**/**kaggle**.**json  !kaggle datasets list |
|  |  |
| **05.** | **Create a directory to save models** |
|  | !mkdir saved_models |
|  |  |
| **06.** | - 1. **Change current directory to project directory.**   **%**cd Localization**-**of**-**Lung**-**abnormalities**-**on**-**Chest**-**X**-**Rays**-**using**-**self**-**supervised**-**equivariant**-**attention  !mkdir data |
|  | - 1. **Download the nih-chest-xrays dataset as a data.zip file into project directory.**   **%**cd data  !kaggle datasets download **-**d nih**-**chest**-**xrays**/**data |
|  | **6.3 Unzip the data.zip file.**  !unzip \***.**zip **&&** rm ***.**zip  **%**cd **..** |
|  |  |
| **07.** | **Install necessary packages** |
|  | !pip install **-**r requirements**.**txt |
|  |  |
| **08.** | **Login to Weights and Biases (wandb) for experiment tracking** |
|  | !wandb login |
|  |  |
| **09.** | **Next, in weights and biases, create a new project titled "chest-xray-localization". Run the trainpcm.py script with the following options.**   1. **--savedirectory : Path to save directory** 2. **--model : Type of model (resnet50, resnet50PCM, efficientnetb4, efficientnetb4PCM)** 3. **--name : Name of current run** 4. **--project : Name of wandb project** 5. **--entity : Name of wandb entity (username) (for more info**[**link**](https://docs.wandb.ai/ref/python/init)**)** |
|  | **%**cd **/**content**/**Localization**-**of**-**Lung**-**abnormalities**-**on**-**Chest**-**X**-**Rays**-**using**-**self**-**supervised**-**equivariant**-**attention  !python scripts**/**trainpcm**.**py **--**savedirectory **/**content**/**saved_models **--**model resnet50PCM **--**name resnet50PCM_v1 **--**project chest**-**xray**-**localization **--**entity YOUR_WANDB_USERNAME |
|  |  |
| **10.** | **During Training, two versions of the model are saved. Final (*FW*) and Checkpoint (*CW*). Checkpoint refers to the model with highest Validation AUROC Score. Go to wandb to view results.**   1. For classification, see AUROC_MACRO_TEST_Checkpoint in summary section. 2. For localization, see table titled RUNNAME-CW-default/pcm/pcm-rv_Checkpoint |
